# From Trust in Automation to Decision Neuroscience: Applying Cognitive Neuroscience Methods to Understand and Improve Interaction Decisions Involved in Human Automation Interaction

**DOI:** 10.3389/fnhum.2016.00290

**Published:** 2016-06-30

**Authors:** Kim Drnec, Amar R. Marathe, Jamie R. Lukos, Jason S. Metcalfe

**Affiliations:** ^1^Human Research and Engineering Directorate, U.S. Army Research LaboratoryAberdeen, MD, USA; ^2^Advanced Concepts and Applied Research Branch, Space and Naval Warfare Systems Center PacificSan Diego, CA, USA

**Keywords:** trust in automation, interaction decisions, decision making, human automation interaction, neuroergonomics

## Abstract

Human automation interaction (HAI) systems have thus far failed to live up to expectations mainly because human users do not always interact with the automation appropriately. Trust in automation (TiA) has been considered a central influence on the way a human user interacts with an automation; if TiA is too high there will be overuse, if TiA is too low there will be disuse. However, even though extensive research into TiA has identified specific HAI behaviors, or trust outcomes, a unique mapping between trust states and trust outcomes has yet to be clearly identified. Interaction behaviors have been intensely studied in the domain of HAI and TiA and this has led to a reframing of the issues of problems with HAI in terms of reliance and compliance. We find the behaviorally defined terms reliance and compliance to be useful in their functionality for application in real-world situations. However, we note that once an inappropriate interaction behavior has occurred it is too late to mitigate it. We therefore take a step back and look at the interaction decision that precedes the behavior. We note that the decision neuroscience community has revealed that decisions are fairly stereotyped processes accompanied by measurable psychophysiological correlates. Two literatures were therefore reviewed. TiA literature was extensively reviewed in order to understand the relationship between TiA and trust outcomes, as well as to identify gaps in current knowledge. We note that an interaction decision precedes an interaction behavior and believe that we can leverage knowledge of the psychophysiological correlates of decisions to improve joint system performance. As we believe that understanding the interaction decision will be critical to the eventual mitigation of inappropriate interaction behavior, we reviewed the decision making literature and provide a synopsis of the state of the art understanding of the decision process from a decision neuroscience perspective. We forward hypotheses based on this understanding that could shape a research path toward the ability to mitigate interaction behavior in the real world.

## Introduction

The purpose of this review is to address a largely unexplored aspect of human automation interaction (HAI); that is, the human decision that leads to interaction behavior, traditionally considered a manifestation of the user’s level of Trust in Automation (TiA). The extension of this concept has been that, if HAI is to be actively managed in joint human-automation systems, one must calibrate the TiA of the user so that decisions about interactions with automation are appropriate. Further, it has been considered that if one could measure instantaneous levels of TiA, inappropriate interaction decisions could be predicted and mitigated. Research interest in HAI systems is motivated in large part because of observations that even the most advanced HAI systems have not yet fully realized the ultimate vision of both safe and seamless integration of the human into the system that would lead to improved task performance. Specifically, successful applications of automation within task spaces involving human operators have not yet been realized without simultaneous definition of significant context-specific design constraints that delineate human and automation responsibilities. Such constraints may improve focused aspects of performance, but also increase the risk in other ways, particularly in circumstances and moments involving handoff of control authority, and these constraints limit more generalized application of HAI concepts and methods, particularly in terms of improving joint system efficiency (Parasuraman and Riley, 1997; Dekker and Woods, 2002; Dzindolet et al., 2003; Jamieson and Vicente, 2005; Parasuraman and Manzey, 2010).

Decades of human factors research have resulted in an understanding of what factors affect TiA, but as of yet, it remains unclear how specific levels of TiA translate into specific human decisions regarding interaction with a given automation. This knowledge gap may exist because human behavior and joint system performance can be thought of as the result of a combination of many factors only one of which is TiA (Hancock et al., 2011; Schaefer et al., 2014). Research aimed at predicting interaction behaviors has previously met with some success, particularly with respect to decision aid systems (Bliss et al., 1995; Meyer and Bitan, 2002; Meyer et al., 2014) and automated driving aids (Kumagai et al., 2003; Gold et al., 2015; Terai et al., 2015). We consider such results to suggest that understanding the interaction behavior may be a more fruitful and immediate route toward active, online mitigation of problems thought to arise from mis-calibrated TiA. This idea is developed with the appreciation that interaction behaviors result from decisions about how and when to interact, and any individual interaction decision may or may not be motivated by a change in TiA.

Our specific proposal is that, as much as behavior is a key to managing HAI, understanding the process of decision-making in the context of HAI is critical for understanding and predicting interaction behaviors. It is an important step that is implicitly necessary for eventual online mitigation of inappropriate interaction behavior. This is especially applicable in our discussion inasmuch as we believe that TiA reflects changing degrees of perceived risk and uncertainty and is an instance of value based decision making in dynamic contexts. We further suggest that physiological correlates of value based decisions could be measured and leveraged to provide valuable data that may increase the likelihood of predicting a consequent interaction. To develop the connection between TiA and value based decision making, the discussion begins by reviewing extant human factors literature to demonstrate that, while TiA is one of many important factors influencing HAI performance, it is ultimately the interaction behavior that is of interest. It is then argued that this behavior, if intentional, is the result of a decision, and thus understanding the decision process leading to the behavior may facilitate near- to medium-term solutions for active mitigation strategies while understanding of the nuances and complexities of TiA continues to evolve over the long term. The discussion then turns towards a synthesis of selected cognitive neuroscience literature that focuses particularly on value based decision making. We conclude with future research directions that would be necessary to enable decision based monitoring and prediction of interaction behaviors and the eventual development of active mitigations for the types of HAI problems currently believed to be brought about by mis-calibrated TiA.

## The Importance of TiA in Joint System Performance

**Figure 1:**
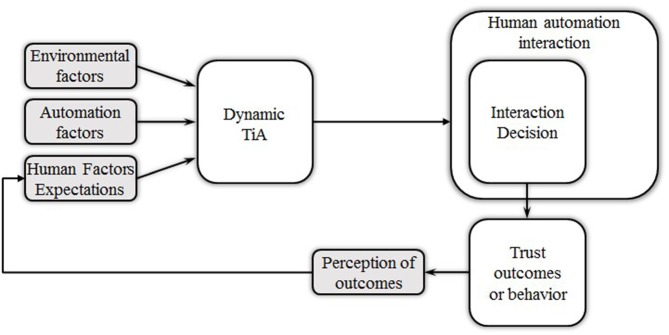
**A conceptual organization of trust and human user-automation interaction (Adapted from Hancock et al., 2011 with permission from Sage Publishing).** This article focuses on interaction decisions that are part of the overall human automation interaction (HAI).

The term automation, or automated system, as used here is best defined by Parasuraman et al. (2000) as a “machine execution of functions”. This definition includes automation with capabilities as diverse as controlling a sophisticated cockpit system or as simple as an automated coffee maker. Because automation is not yet fully “intelligent” it has no agency for adapting to unexpected circumstances, and therefore often requires the supervision and/or occasional intervention of humans. Part of this supervisory role requires that there be HAIs, but these interactions need to be appropriate, or the joint system performance will suffer. Decades of HAI system research have indicated that appropriate interactions are the result of decisions subsequent to calibrated TiA. An established conceptual model of factors influencing HAI performance is provided in Figure 1. As the model suggests, TiA has traditionally been considered to be the critical component driving human user decisions about interactions such as intervening in an automated task. Given the importance that TiA has been accorded to overall joint system performance, in this section we provide a brief review of important aspects of TiA and its dynamics. We aim to highlight the complex relationship between TiA and human user behavior, and implications arising from this relationship that imply that even if moment to moment levels of TiA were to be measured, it is unclear how such information could be leveraged to predict an interaction behavior.

Early theories about the construct of TiA were developed from the psychological construct of interpersonal trust, and they posited that calibrated TiA was critical for successful HAI system performance (Sheridan, 1980; Sheridan and Hennessy, 1984). There are aspects of interpersonal trust that are analogous to TiA, in particular that there needs to be a sense of risk or vulnerability on the part of the trustor for trust to develop (Lee and Moray, 1994; Muir, 1994; Corritore et al., 2003; Lee and See, 2004; Evans and Krueger, 2011). However, it has been debated whether the two constructs are homologous (Madhavan and Wiegmann, 2007), and so trust as it specifically applies to automation became a central point of interest in human factors research aimed at improving joint system performance (Lee and Moray, 1992; Muir, 1994; Muir and Moray, 1996; Lee and See, 2004). Myriad definitions of TiA imply that it is the result of a feeling of trustworthiness towards the automation such that a human user can depend on the automation to perform the task for which it was designed. It is worth noting that if the consequence of the task to the human user is small, if TiA develops at all, its level becomes irrelevant because the outcome of the joint system fails to be important. Therefore, much like interpersonal trust (Lee and Moray, 1994; Muir, 1994), TiA develops in the face of a sense of risk. In these situations, TiA then develops and shows dynamic changes from the ongoing comparison of the expectations about the automation’s behavior and observations by the human user about the automation’s performance weighted heavily on the risk borne by the human user (Sheridan and Hennessy, 1984; Muir, 1994; Muir and Moray, 1996).

### Determinants and Dynamics of Trust in Automation: Expectations and Observations

One of the first explicit theories of TiA (Muir, 1994) stated that appropriate levels of TiA would develop if three expectations were met during the course of automation interaction. These expectations are technical competence, persistence, and fiduciary responsibility, but they play differential roles in TiA development and dynamics throughout the course of automation use. For example, perceptions of competence might be more important in the early stages of automation use than later in time. The expectation of technical competence is the expectation that the automation will accurately and successfully perform the functions for which it was designed. Persistence, perhaps here better conceived of as predictability, relates to the issue of reliability in that an automation that performs in a particular manner now will be expected to perform in a same or similar manner when it encounters similar circumstances in the future. Finally, fiduciary responsibility addresses the notion that a given human user will hold expectations of an automation of a particular type that will impact role allocation. That is, the human user will expect that the automation will necessarily be responsible for its designed functions as they understand them and thus fewer personal resources need to be allocated to carrying out those functions. The importance of these expectations related to TiA dynamics differ depending on the stage of the interaction with the automation.

When first presented with an automated system there is limited information available for the human user to observe, and thus little with which to evaluate the trustworthiness of the automation. Some key elements that significantly affect early levels of TiA include initial expectations borne from biases toward automations in general, and initial observations about the design of the automation (Muir and Moray, 1996; Nass et al., 1996; Dzindolet et al., 2002; Lee and See, 2004; Parasuraman and Miller, 2004; Miller, 2005; Merritt and Ilgen, 2008; Merritt, 2011; Merritt et al., 2012; Pak et al., 2012). After having been introduced to an automation, human users tend to explore different strategies for subsequent interaction (Lee and Moray, 1992) and thus learn more about the automation’s behavior. This experimentation arguably helps the human user gauge competence, which emerges as one of the most important predictors of TiA at this early stage. However, it is worth noting that for various reasons, human users are notoriously poor at making accurate judgments of competence (Sheridan and Hennessy, 1984; Lee and Moray, 1992; Dzindolet et al., 2002, 2003; Madhavan et al., 2006; Verberne et al., 2012; Merritt et al., 2014). Once automation competence has been judged, whether correct or not, the most important factor driving levels of TiA is persistence or predictability of performance over time (Lee and Moray, 1992). Persistence of performance is important enough that as long as errors are predictable and the automation error rate is at a consistent rate of approximately 30% or less, most human users will decide to continue to use and benefit from the automation (Parasuraman et al., 2000; Wickens and Dixon, 2007; Wang et al., 2009). As levels of TiA dynamically change throughout the course of observations about the automation’s behavior, theory posits that interaction decisions, and consequent behaviors, should reflect the extant level of TiA. If there is too much or too little TiA, as it goes, a human user may decide to overuse or underuse the automation, respectively. Specific patterns of behaviors resulting from decisions about how to interact with the automation have been well documented and are commonly referred to as trust outcomes as they are believed to directly reflect certain levels of TiA.

### Trust Outcomes and their Relationship to TiA

The trust outcomes most commonly discussed are misuse and disuse and are described in detail by Parasuraman and Riley (1997). Misuse refers to instances when the automation is used without undo skepticism, tending to result in overuse (Parasuraman and Riley, 1997; Bahner et al., 2008; Parasuraman and Manzey, 2010). Misuse has two related causes; automation bias and complacency (Manzey et al., 2006; Parasuraman and Manzey, 2010). They are related in that they both result in a lack of monitoring where lack of attention plays a central role (Parasuraman and Manzey, 2010). Automation bias arises through the mere presence of an automated system, possibly because humans demonstrate a tendency to choose the route of least cognitive effort, making it easier, or at least preferable, to accept that feedback from an automation as correct (Dzindolet et al., 1999, 2001; Skitka et al., 1999, 2000; Wang et al., 2008; Parasuraman and Manzey, 2010; Goddard et al., 2014; Mosier and Skitka, 1996). Complacency, less well understood, can be said to occur when monitoring is less than optimal and joint system performance suffers (Parasuraman and Manzey, 2010). However, both automation bias and complacency tend to increase in cases of high workload and high consequence environments wherein users often make conscious decisions to rely on even imperfect automation (Dixon et al., 2007; Wickens and Dixon, 2007). Disuse describes a continuum that spans from the user underutilizing the automation to entirely abandoning the automation in favor of a manual mode. Disuse tends to occur if a human user has a high expectation of automation performance and then observes unexpected errors or has more self confidence in her ability to perform the task than confidence in the efficacy of the automation for the same task (Lee and Moray, 1994; Parasuraman and Riley, 1997; Moray et al., 2000; Dzindolet et al., 2003).

Although trust outcomes have been well defined, a synthesis of the literature, and recent experimental evidence (Wiczorek and Manzey, 2010; Chancey et al., 2015) indicate a far more complex relationship between TiA and trust outcomes than is implied in the above discussion, and this implies that predicting interactions based on extant TiA levels is problematic. Such complex interactions involve perceived risk, self-confidence, workload, and even personality type (Lee and Moray, 1994; Muir, 1994; Parasuraman and Riley, 1997; Lee and See, 2004; Merritt and Ilgen, 2008; Hancock et al., 2011; Schaefer et al., 2012; Merritt et al., 2014). For instance, human users have been documented as reporting a high level of TiA and then, paradoxically choosing a manual operation mode, demonstrating disuse (Lee and Moray, 1992). Conversely, even when low TiA has been reported, human users may misuse even a poorly competent automation, particularly under high workload conditions (Daly, 2002; Biros et al., 2004). Clearly levels of TiA do not map uniquely onto trust outcomes, regardless of how they are represented (i.e., attention, intervention rate, etc.). Therefore, they are not predictive of the way a human user will decide to interact with an automation, limiting the use of measuring TiA for real world applications to improve HAI. We suggest that this is because TiA is far more complex than may be useful for those with more immediate concerns regarding actively managing HAI. However, it is important to note that when TiA is studied it is the interaction behavior that is of interest most often. Therefore, regardless of the manageability of trust, what might be learned if we focus more simply and exclusively on the behavior?

### TiA as Predictable Behavior

The present discussion is not the first to offer that a shift in focus from trust to behavior is well justified. In fact, a number of researchers in this domain have re-framed the problem space of TiA into one of reliance and compliance, which are defined exclusively in terms of observable behavior, and are not intended to imply specific psychological cause such as trust (Meyer, 2001, 2004; Parasuraman et al., 2008; Rice, 2009; Meyer et al., 2014). Indeed, there is a non-unique mapping of reliance and compliance to traditional trust outcomes such that an observation of inappropriate amounts of either may alternately signal disuse or misuse and possibly motivate conflicting interpretations of TiA. We find these behaviorally defined terms to be useful in their functionality for application in real world situations. That is, objectively defined and observable behaviors are especially valuable for the purposes of modeling and prediction because they obviate the need for drawing inferences to and making assumptions about manifestations of more subjectively defined constructs, such as TiA, automation bias or complacency that are difficult to measure objectively and thus unsuitable for use in attempts at active optimization and/or mitigation.

Reliance is the tendency of the human user to accept the lack of an alarm, alert, warning, or prompt as a true reflection of the state of the world (Lee and Moray, 1994; Singh et al., 1997; Parasuraman et al., 2000; Yeh and Wickens, 2000; Moray, 2003; Dixon et al., 2006). That is, in the absence of an alarm or warning, the human user accepts, often tacitly, that all is well and there is no reason for possible intervention. Compliance, on the other hand, is defined when the user responds to, putatively agrees with, and ultimately takes the action specified by an alarm or recommendation from the automation (Meyer, 2001, 2004). Though reliance and compliance are often discussed in terms of optimal behavior, too much of either in the wrong context is detrimental to system performance. For instance, if an alarm is absent and the human user assumes that no circumstances warranting an alarm exist and thus fails to monitor the automation over significant time, he or she is at risk of over-reliance and the consequences thereof. Conversely, over-compliance occurs when the human accepts all suggestions from the automation (when present) without confirming their validity.

Beyond observation of general behavioral patterns, the greater benefit of defining compliance and reliance behaviors has been in providing an avenue towards greater precision in understanding the factors that affect automation use during HAI, which might eventually lead to prediction of an interaction. For instance, some have observed that reliance and compliance are differentially affected by error type, i.e., false alarms vs. misses in target detection tasks (Meyer, 2004; Rice and Geels, 2010; Wiczorek and Manzey, 2014), and by the predictive value of the alarm (Meyer and Bitan, 2002; Manzey et al., 2014). If a human user observes frequent failures to trigger alarms, the frequency of monitoring the automation will increase, thereby reducing reliance on the automated agent (Masalonis and Parasuraman, 1999; Bagheri and Jamieson, 2004; Meyer, 2004; Madhavan and Wiegmann, 2007; Parasuraman and Manzey, 2010; Geels-Blair et al., 2013). Compliance, however, is degraded by higher rates of false alarms. In particular, when higher rates of false alarms are observed, users tend to consume critical time and attentional resources to verify alarms before choosing a response. Further progress in this line of inquiry has resulted in more general characterization of how interaction behaviors change with the positive and negative predictive value of an alarm. Positive predictive value is derived from a Bayesian calculation of the likely existence of a hazard given an alarm and, likewise, the probability of an alarm given no existing hazard (Meyer et al., 2014). Negative predictive value is calculated similarly, but in the absence of an alarm. Therefore, positive predictive value decreases as false alarm rate goes up and negative predictive value decreases with more frequent misses (no alarm in the presence of a hazard). Interaction behavior is thus differentially affected by changing positive vs. negative predictive value. Positive predictive value has been shown to have strong effects on reliance, but only for values less than 0.75 (Meyer et al., 2014), where values below this threshold have been associated with excessive time spent monitoring the automation. Research in HAI domains has thus befitted considerably from the use of these narrowly and objectively defined behavioral terms.

We advocate here for shifting research towards more clearly defined behaviors and the factors that affect them because of how this shift creates important opportunities for systematic research into HAI. The domain of application for such a shifted focus would include contexts where TiA may be involved, at least inasmuch as TiA reflects assessments of the relative value of specific behavioral options defined in terms of probable risk versus reward. We argue that such behavior-based understandings are important for progress on multiple levels from phenomenology to predictive modeling. The extant work discussed above has provided an essential corpus of knowledge regarding the relationship between automation performance characteristics (i.e., error rate, type, and predictive value) and human user interaction behaviors. However, we also suggest that in order to be useful down the road for real-world mitigation of inappropriate interactions, this shift from trust to behavior does not go quite far enough for two important reasons. First, to mitigate a potentially detrimental interaction behavior in a dynamic context, prediction is necessary. This is because a behavior that has already occurred cannot be changed and the consequences are likely to be too immediate to offset in *post hoc* fashion. Moreover, the predictive power required must occur on a time-scale that allows a reasonable opportunity to enact a mitigation when an inappropriate behavior is expected. Second, the current understanding of reliance and compliance is tied to automation design; an automation that frequently misses events reduces reliance, and an automation that frequently produces false alarms reduces compliance. This understanding, then, usefully provides an improved framework for HAI, but has yet to account for variability in individual instances of HAI. Therefore, the predictive power of the current understanding of interaction behaviors based on population averages remains limited to overall design strategies whereas we are interested in building towards eventual prediction and mitigation of reliance and compliance at the level of individual instances of interaction behavior. In order to improve the ability to predict an interaction behavior we thus believe it is necessary to consider not only the effects of automation design on interaction behaviors such as compliance and reliance, but also the individual internal phenomena that precedes the behavior.

## Interaction Behavior Reframed as a Decision to Interact with an Automation

Before an intentional behavior occurs, the human user must make a decision as to which among a limited array of options will be selected. Here we argue that research concerned with improving HAI would benefit greatly from studying the decisions that precede interaction behaviors. Such an approach satisfies the need for focus on individual interactions in a manner that affords prediction on a time-scale that is useful for active mitigation. We define such decisions as interaction decisions, given as specific to the intention to interact with an automation. While TiA has often been considered to motivate interaction decisions, the richness of the decision process itself, as well as accompanying stereotypical psychophysiological indicators thereof, has not been thoroughly investigated as a source of information that could be applied to the prediction of a consequent interaction behavior. Our starting point in this pursuit is to understand the underlying psychological and physiological processes of decision making, with a particular focus on value based decision making. This understanding can provide a cornerstone for the advancement of scientifically based hypotheses about how interaction behaviors may eventually be predicted for the sake of active mitigation. Predicting decision outcomes, or the interaction behavior in a real-world HAI context, is of course not trivial, but laboratory-based research in decision neuroscience has established decision making as a reasonably stereotyped process with clear behavioral and physiological precursors. Further, the ability to predict decision outcomes has been pursued by both the cognitive neuroscience (Soon et al., 2008; Haynes, 2011; Perez et al., 2015) and brain computer interface (Musallam et al., 2004) communities. Indeed, attempts at predicting some types of decision outcome behaviors have already met with success in a laboratory environment, possibly because the specifics of a decision process in the brain begin even before there is conscious awareness of the impending decision (Soon et al., 2008, 2013; Haynes, 2011; Perez et al., 2015). Some of these studies have been criticized because there lacks a sense of risk or value to the decision maker in a controlled experiment, and therefore, the assumption is that the decision outcomes that are being predicted are trivial (Gold and Shadlen, 2007; Lavazza and De Caro, 2010).

The lack of risk, value, or reward in these controlled laboratory environments is in contrast to interaction decisions that inherently involve some type of personal risk or reward. For example, over relying on an automation can compromise joint system performance, and therefore causes degradations in joint system performance. Thus, we are chiefly concerned with decisions that are based on expected value and risk, or value based decisions (Rangel et al., 2008; Wallis, 2012). Value based decisions are particularly relevant to the HAI context because it is often required that a human user continuously weigh the expected personal value of allowing the automation to complete the task versus performing it manually or, rather, whether to comply with the recommendation of an automated system. Importantly, this assessment and subsequent judgment of value to the user must be made against the backdrop of risk that the decision may compromise joint system performance. Thus, through the common elements of risk, reward, and expected value, we believe interaction decisions during HAI to be an instance of value based decision making. We believe that understanding the value based decision process is important to improving HAI and, therefore, we briefly discuss results of value based decision making research as it relates to HAI in order to support hypotheses forwarded in the discussion, aimed at establishing a research path that will allow the eventual prediction of HAI interaction behaviors.

### The Importance of Considering the Decision Process

An important argument in favor of studying the decision process in order to improve HAI is that significant efforts in cognitive neuroscience have revealed decision making as fairly stereotyped, and therefore, a potentially predictable process. Moreover, this body of research has identified a number of psychophysiological correlates that unfold in advance of, and during a decision. Critically, these correlates are measurable, and therefore useful for understanding the decision process, at least in laboratory settings. Some have observed that these correlates unfold in predictable ways through defined cognitive stages, and therefore measuring them has potential use for active mitigations of inappropriate interaction decisions and behavior. This approach is fundamentally different than attempting to measure and calibrate TiA because the psychophysiological correlates of a decision are measurable whereas the construct of TiA is yet to be defined in a way that is equally useful for active monitoring. In general, many cognitive neuroscientists model decisions as comprising three cognitive stages (Fellows, 2004; Bogacz, 2007). However, five cognitive processes, some analogous to stages of the cognitive models of general decisions, have been described in value based decision making (Rangel et al., 2008) and are therefore relevant to our discussion of interaction decisions. These processes, which are not discrete stages *per se*, are: (1) representation of the problem, i.e., identification of alternative choices, and of internal and external states that affect the value of the choices; (2) evaluation of gathered evidence that allows the assignment of a value to the alternatives; (3) comparison of these values in order to make a decision; (4) accumulation of the comparative value for each alternative and making the decision; and (5) generation of prediction errors that provide feedback in order for learning to occur. The psychophysiological processes that unfold within the first four processes will be discussed as they relate to HAI contexts such as risk and reward. The fifth process, generating feedback on the decision has been studied in the context of learning, and may be useful for later development of adaptive mitigation strategies, but is beyond the scope of the current review (Nieuwenhuis et al., 2005; Christie and Tata, 2009; Cohen et al., 2011; van de Vijver et al., 2011). We note that we discuss these processes sequentially mainly for organizational purposes, however, during the decision process they may overlap or even occur in parallel (Rangel et al., 2008).

### The Value Based Decision Process

In order for the need to identify alternatives to arise, there must be some recognition of the need for a decision; in a sense it is the motivation to perform a task (Gold and Shadlen, 2007). Decisions must be initiated by either salient external or internal stimuli. These stimuli will often produce an orienting response (Sokolov et al., 2002; Glimcher and Rustichini, 2004; Delgado et al., 2005), characterized in humans by a measurable increase in tonic skin conductance (SC) levels and a decrease in heart rate variability (Figner and Murphy, 2011). In an HAI domain, relevant stimuli typically include those specifying alerts from the automation, acute changes in environment, or internal feelings that the current behavior is inappropriate (typically seen as an error-related potential in the brain or a gradual shift in peripheral physiology). Once the need for a decision has been established, however, decision alternatives are identified. As alternatives are identified, in the case of interaction decisions, the human user will also identify, if not consciously, a representation of internal and external states (Rangel et al., 2008). These representations play an important part during the process of assigning values to individual alternatives. For example, a human user is more likely to take control from the automation if they detect that the automation is malfunctioning and they perceive an associated risk. The neural basis of this early stage in the decision process is not well understood. For example, it is unclear how the brain decides which alternatives should be considered, and if there is a functional limit to the number that can be assessed at one time (Rangel et al., 2008). Nevertheless, such questions are important for determining how to leverage physiological indicators into models of decision making during HAI.

Once the possible alternatives are identified evidence for or against each alternative must be evaluated in order to make an optimal decision. In the case of interaction decisions, which due to the presence of risk are analogous to value based decisions, it has been hypothesized by some (Rangel et al., 2008; Glimcher and Fehr, 2013) Reading hidden intentions in the human brain cognitive valuation systems that the brain might use; the Pavlovian, Habitual, and Goal directed. We believe the goal directed system to be most relevant to our discussion because the goal directed system assigns values to potential actions by calculating action-outcome associations from previous experience and comparing this value to the perceived rewards associated with possible outcomes of the decision (Rangel et al., 2008). In the goal directed valuation system the value assigned to a piece of evidence is equivalent to the potential value of the alternative it supports, with the value assigned to an alternative being equal to the expected reward of the action. In the context of HAI, an important research question would be whether the probability of success is greater by relying on the automation or not and, moreover, how that probability scales with perceived risk to determine the direction of a given interaction decision.

When a person valuates a piece of evidence they will do so by observing the relevant data (e.g., visual scanning, sound or other stimulus), consulting their memory, and integrating this against a backdrop of expectations (Mulder et al., 2014). In the case of visual evidence, gaze fixation is thought to support evidence evaluation such that evidence about the value of an alternative is sampled at each fixation (Krajbich et al., 2010; Krajbich and Rangel, 2011). Memory consultation causes a person to compare past decision outcomes with the available alternatives. The brain creates a prediction error that would represent the difference between the expected value of choosing current decision alternatives from the value that has been experienced in the past by choosing alternatives that are similar in nature (Hare et al., 2008). For example, consider a human user who has previously experienced aberrant behavior from an automation, but there has been no decrement in joint system performance, and joint system performance continues to remain better than what would be expected from only one agent performing the task. Even in risky environments such as in the battlefield, or during a search and rescue operation, the experienced user is more likely to rely on an automation (Lyons and Stokes, 2012) than a user who has not experienced the aberrant behavior because the experienced user has realized the value in relying on the automation despite the probability of an error.

At a cellular level, data have suggested that the cognitive evaluation of evidence is supported by neural “evaluators” that store dynamic estimates of which decision alternative is supported by the evidence. For instance, studies using fMRI in risk reward scenarios have identified two candidate neural evaluators; the amygdala and ventral striatum. A reward based fMRI study indicated that the amygdala evaluates the cost or risk of acting on an alternative (Yacubian et al., 2006; Basten et al., 2010). In the same fMRI study the ventral striatum was implicated in the formation of representations of the expected value or reward of an alternative (Yacubian et al., 2006; Kable and Glimcher, 2007; Rangel et al., 2008; Basten et al., 2010; Lim et al., 2011). Other authors, however, have found that in addition to the amygdala and the ventral striatum that the lateral orbitofrontal cortex and the medial orbital frontal cortex also act as neural evaluators for risk and reward, respectively (Hare et al., 2008; Rangel et al., 2008; Rangel and Hare, 2010). The neural substrates that have been observed support value based decision making processes are detailed in Table 1.

**Table 1:** **Neural substrates and their putative role in decision making**.

Neural substrate	Role in decision making	Reference
Amygdala	Processes/computes the value of negative stimuli	Yacubian et al. (2006) and Basten et al. (2010)
Ventral striatum	Processes/computes the value of positive stimuli	Yacubian et al. (2006); Basten et al. (2010) and Lim et al. (2011)
Ventral medial prefrontal cortex (vmPFC)	Calculates the difference of value signals from amygdala and ventral striatum in value based decisions	Basten et al. (2010) and Philiastides et al. (2011)
Dorsolateral prefrontal cortex (dlPFC)	Calculates the difference of signals from amygdala and ventral striatum in perceptual decisions	Basten et al. (2010) and Philiastides et al. (2011)
Lateral intraparietal cortex (LIP)	Accumulates and integrates the value of evidence processed by the vmPFC (evidence largely from monkeys)	Platt and Glimcher (1999); Platt (2002); Basten et al. (2010) and Rorie et al. (2010)
	A cortical area involved in gaze fixation, saccade, and attention, underlying evidence accumulation	Coe et al. (2002) and Goldberg et al. (2006)

The neural evaluators, then, form representations of the risks and rewards for each alternative; the benefit of relying or complying with an automation, as opposed to choosing to complete the task manually. As the risks and rewards of a potential interaction behavior are processed by the amygdala and ventral striatum, the value of these representations must be assessed relative to each other; they must be compared. Neural correlates of this third process involved in value based decision making, comparison of the values assigned to the evidence, have been observed in fMRI studies. That is, value based comparison has been suggested as supported by activation in the ventral medial prefrontal cortex (vmPFC; Chib et al., 2009; Gläscher et al., 2009; Basten et al., 2010), whereas the “comparator” function in perceptual decisions has been associated with increased activity in the dorsolateral prefrontal cortex (dlPFC; Basten et al., 2010; Philiastides et al., 2011). While the evidence comparison process unfolds, some have hypothesized that the comparative value, also known as the decision variable, is accumulated in the lateral intraparietal cortex (LIP) until a decision threshold is reached, bringing about a decision (Platt and Glimcher, 1999; Kiani and Shadlen, 2009; Mulder et al., 2014). Evidence to support this hypothesis has mainly been shown in primate studies of single cell recordings during cued saccade trials (Platt and Glimcher, 1999; Platt, 2002). However, there has been evidence from fMRI studies that the human parietal cortex is also involved in accumulating the decision variable (Ploran et al., 2007; Heekeren et al., 2008). It is interesting to note that the temporal integration of activity in the frontal-parietal regions, which are considered to be involved in comparing and accumulating compared value signals, has been observed as preceeding the conscious decision to act (Gold and Shadlen, 2007; Soon et al., 2013; Perez et al., 2015).

The putative involvement of the parietal cortex in decision making is noteworthy because of its central role in the process. For example, gaze fixation, critical for evidence evaluation (Poole and Ball, 2006) in visually based decisions, is controlled by the LIP in monkeys (Coe et al., 2002). This region forms a “salience map” for the oculomotor system to saccade to a target, or maintain gaze fixation on a target (Goldberg et al., 2006). The LIP then, not only plays a role in accumulating the comparative value of the evidence as discussed above, but is critical for its initial evaluation. Brain computer interface research has also found that the medial intraparietal cortex in monkeys forms representations of the value of an alternative that has been encoded in the vmPFC (Musallam et al., 2004), such that the intent of the monkey to choose one alternative over an other can be decoded from intracellular electrodes. Although much evidence of the importance of the parietal cortex during decision making, and especially value based decision making, has come from primate research, there is evidence that analogs in the human parietal cortex are also central to decision making (Ploran et al., 2007; Heekeren et al., 2008).

Despite the hypothesized causal role of integrated frontal-parietal activity in the conscious decision to act, there are no measurable psychophysiological variables that allow an accurate determination of the exact time that a decision threshold is reached. However, psychophysiological correlates occurring hundreds of milliseconds before a conscious decision involving risk and reward (Cohen et al., 2009), inherent in value based decision making, have been identified. For instance, the readiness potential, a slow negativity in scalp recording of cortical activity precedes fully endogenous decisions by a few hundred milliseconds (Libet, 1993). Even more proximal to the decision spectral correlates have been observed. In a paradigm involving playing a competitive game against a computer, spectral decomposition of scalp-recorded EEG led to the finding that the decision process was accompanied by a general shift in power between lower bands (delta, 1–4 Hz and theta, 5–7 Hz) to higher frequency bands (alpha, 8–12 Hz and beta, 13–35 Hz), as well as a broadband increase in cross-trial phase coherence at about 220 ms post stimulus (Cohen and Donner, 2013). Similar indications were found during complex real world choice tasks and a two-choice forced-decision paradigm. In these cases, significant correlations of increased power were seen in delta, theta, beta, and gamma (36 + Hz) bands of EEG activity approximately 250–500 ms post-stimulus (Guggisberg et al., 2007; Davis et al., 2011). In decisions involving risk, risk is represented by an asymmetry in the alpha band such that there is an increased alpha power in the right frontal region (Gianotti et al., 2009). These spectral correlates of value based decision making are measurable in real time and available for current application outside a laboratory, which is encouraging in the context of improving HAI. However, these scalp recorded spectral correlates occur only hundreds of milliseconds before an interaction decision and therefore have limited use because they are so temporally proximal to the behavior itself.

The proximity of these value based decision correlates to the actual decision may be discouraging in the context of predicting and mitigating interaction behavior. Nonetheless, research efforts in decision neuroscience and in brain computer interface have found in fMRI studies that the correlates of an outcome of a decision to move at a time chosen by the subject are measurable up to 7 s before the conscious awareness of the decision is reached. Moreover, through analysis of these correlates, the intended goal of the decision can be decoded before conscious awareness of it arises (Haynes et al., 2007; Soon et al., 2008, 2013; Haynes, 2011; Perez et al., 2015). In two fMRI studies (Haynes, 2011; Soon et al., 2013) subjects were asked to decide at will when to either press a button on their left or right side, or add or multiply a set of numbers, and then to report when they were consciously aware of the decision. Spatial pattern analysis of the blood oxygen level dependent signal, a measure of neural activation in fMRI studies, revealed that the frontal polar cortex appeared to encode the intentions of the subjects before they reported having made the decision. In a driving study using implanted EEG electrodes in human epilepsy patients a modulation of gamma power in the posterior lateral cortex predicted whether the subjects would turn left or right at an intersection before they consciously made the decision (Perez et al., 2015). These studies made use of technology such as fMRI that as yet is not available for real world applications, unlike EEG, because of the need for the subject to lie still in the large, importable fMRI equipment. Further, although EEG is portable, it cannot directly measure activity in deep cortical and subcortical areas, and these are exactly the areas that showed activity prior to a conscious decision. However, the results are encouraging for making use of the interaction decision process to improve HAI and, moreover, can be leveraged into research aimed at developing models that capture relations between cortical and subcortical brain activations during value based decision making. Identification of such relations is an important avenue for future research aimed at active mitigation during HAI.

Indeed, decision neuroscience and brain computer interface research have facilitated the development of precise understandings of decision making that could facilitate the development of methods for identifying an interaction decision within the contextual space of HAI. Efforts in decision neuroscience as well as in more applied domains, such as neuroergonomics, have shown that there are clear and measurable behavioral and psychophysiological correlates (fMRI activation patterns, EEG, SC, gaze fixation, heart rate, etc.) of component processes that are antecedent to the decision. In addition, decision neuroscience has begun to provide an understanding of the underlying cortical and sub-cortical processes involved in decision making. While many of these processes have as yet only been identified by fMRI, their understanding will allow meaningful hypotheses to be advanced about measures that can be recorded in real time.

## Discussion

HAI systems have as yet to live up to their expectations, and one critical reason is that human users often make inappropriate decisions about how and when to interact with an automation. These interaction decisions have traditionally been considered to be motivated by extant levels of TiA. Therefore, if TiA can be measured, it is expected that it can be managed and inappropriate interaction behaviors could be mitigated. Given that substantive theory, it was appreciated that TiA is an important construct that undoubtedly affects human user interaction behavior, and hence we reviewed and synthesized the TiA literature. From that exercise, we observed that the relationship between TiA and human behavior is complex and not fully understood. Further, relevant to immediate real world applications for improving HAI system performance, TiA cannot be readily measured, and even if it were measurable in real time it is unclear how certain levels of TiA map onto specific interaction behaviors. By contrast, specific behaviors such as reliance and compliance are readily observed and measured in real time and do not have the confounding effect of inferring psychological causality. Such cause-agnostic variables are particularly attractive in HAI research aimed at defining concrete methods for improving joint system performance, both in terms of initial system design as well as for ultimate real-time applications.

Reframing the problem space of HAI and TiA as a problem of behavior, rather than of TiA, has been successful in allowing general predictions of interaction behavior based on knowledge of system design given specific environmental and internal conditions such as increased risk or increased workload, respectively. For example, knowing that an automation is prone to false alarms will allow the general prediction that a human user will often fail to comply with alerts. This understanding allows a system designer to set thresholds for alarms that are appropriate to intended use. For example, in high risk environments it may be better to set an alarm threshold low so that critical cases are not missed. This predictive ability has been significant in designing systems, but the knowledge is unlikely to allow active mitigation of interaction behavior on an individual basis in real time application, an implicit goal of the aims of HAI focused research. This is the case for two important reasons. First, we argue that focusing attention on interaction behavior does not go far enough because once the behavior has occurred it is too late to mitigate it in an *post hoc* fashion in a timely manner. Second, behaviorally based predictions for automation use are by definition general because the predictive ability has been achieved through extensive observation of how human user behavior is affected by system design. The behaviorally based predictions, however, do not take into account individual variation and dynamic changes in environment. Therefore, they are unlikely to apply to individuals on a case by case basis. For example, it has been shown that a human user may continue to rely on an automation that persistently commits errors of omission, or “misses”, due to automation independent reasons such as increasing workload. Therefore, an approach is needed that considers individual cognitive and behavioral aspects, as well as ensuring that there is time to not only mitigate behavior, but allow the prediction of the likelihood of an interaction behavior.

We note that antecedent to the interaction behavior is a decision, which has been characterized by the decision neuroscience community as fairly stereotyped and accompanied by measurable psychophysiological correlates. These properties of decisions suggest predictability, and importantly, as decisions are individual in nature, these properties also imply the likelihood of behavioral prediction at an individual level. We thus believe that understanding the interaction decision is a useful approach to improving HAI, and that with future research that the decision correlates can be leveraged to predict the likelihood of an individual’s impending interaction behavior. This approach not only satisfies the problems just discussed, but takes into consideration the human and environmental variability that is found in real world situations in ways that research focused on reliance and compliance has yet to achieve. While it is true that many of the psychophysiological correlates of value based decisions are only measurable with fMRI, we consider that the discoveries afforded by fMRI studies provide a solid basis to form specific scientific hypotheses to guide future research aimed at understanding the interaction decision and consequent interaction behavior. We understand that the fruit of decision neuroscience research might be applied to any domain where it would be advantageous for one decision outcome over another. However, our domain of interest is HAI, and therefore, our focus is in leveraging what is known about value based decision making to understand interaction decisions in the hopes of eventually predicting the likelihood of one decision over another. This goal will require future research, and we begin by forwarding hypotheses to guide research efforts.

One of our first assumptions in this review is that interaction decisions are in fact a case of value based decisions, and that assumption guides our first hypothesis; interaction decisions are a special instance of value based decisions, and therefore the neural correlates accompanying value based decisions will be observable during interaction decisions. One of the first avenues of research is to demonstrate that value based decisions and interaction decisions are analogous in that the sense of risk and reward are inherent in both. This could be achieved by measuring bilateral frontal alpha power during an interaction decision to look for the characteristic asymmetry found in situations entailing risk. Further research to support this hypothesis should necessarily include observing the predicted vmPFC-parietal activation found in value based decision making, during an interaction decision. The strength of this evidence, if found, could be enhanced if concurrent with the parietal-vmPFC activation there is significantly less activation of the dlPFC.

If our first hypothesis is confirmed, we can begin to make more specific hypotheses. Our second hypothesis relates to the fact that there is little understanding of the first stage of value based decision making, the observation of alternatives and representation of internal and external states. Understanding this stage could be particularly important for mitigating inappropriate behavior because it is also accompanied by physiological changes (SC, decreased heart rate variability), which are readily observed. We believe that future research should be aimed at revealing neural correlates of this stage. We hypothesize that activity in the frontal polar cortex and in areas of the parietal cortex during, or just prior to a conscious decision to interact with an automation will occur along with or just prior to the physiological correlates. Evidence previously discussed, that activation patterns in the area of the frontal polar cortex in humans, and in the area of the mid-parietal in monkeys, can be decoded to reveal behavioral intention supports this hypothesis. Should this hypothesis be confirmed, it would add significant evidence that the approach of focusing on interaction decisions will provide an improved method to mitigate interaction behaviors on an individual level. For example, consider the fact that users tend to rely on an automation in the face of risk as demonstrated by traditional behavioral research. However, if this interaction behavior is inappropriate, but the decision to rely can be decoded, there is a chance to mitigate the inappropriate interaction behavior.

While not hypotheses, we believe that future research should also be focused on understanding the psychophysiological basis for, or correlates of, the interaction behaviors of relying or complying on an automation. One first step should include finding the psychophysiological correlates of the demonstrated tendencies of users to rely or comply with automations in circumstances such as workload and risk. For example, what psychophysiological processes drive a human user to perhaps over comply with an automation in conditions of increased cognitive workload, and are there measurable correlates that would suggest that this is the likely interaction behavior? Conversely, what psychophysiological correlates can be found, apart from alpha asymmetry, that appear to suggest the human user is perceiving increased risk, and therefore more likely to over rely on an automation? In order to mitigate disuse, potential psychophysiological correlates, such as particular levels of heart rate variability, SC and EEG power require future study.

Finally, though on a longer horizon, we suggest that it might ultimately be feasible to leverage the understanding of value based decisions in behavioral mitigations aimed at improving HAI system performance. Logically, management of a particular interaction between a human and an automated system requires a minimum of three elements. First, it is essential to understand the capabilities and vulnerabilities of both the particular operator and the particular automation as well as how these may vary under different task and contextual constraints. With such knowledge, one may be able to infer an optimal strategy for allocation of control or decision authority, such as has been done with behaviorally based predictions. Second, though the behavior of automated controls is relatively predictable with knowledge of how its control system was designed, establishing likelihood of human behaviors is a much more challenging task. Therefore, it is also critical to develop methods for prediction of likely changes in operator behavior on a time-scale that leaves room for active intervention through the understanding of the interaction decision. Third, an understanding of how to influence the decision process of humans in principled ways is necessary to ultimately define appropriate systems of actuation when inappropriate behaviors are expected. Of these three elements, it seems that the second may be the most challenging. This is because it is relatively trivial to establish baseline operational or performance characteristics of both humans and automated systems and it is already known that human behaviors and perceptions are subject to influence by a variety of factors, including workload, display properties, transparency, and may be amenable to influence by other task and contextual factors. However, predicting impending behavioral choices is particularly challenging because this requires methods to develop advance insight into the unfolding of the decision process that has largely been studied through the use of fMRI. Here, we offer one way of addressing this; by the application of modern techniques from cognitive neuroscience and psychophysiology.

## Conclusion

The main purpose of this review is to explore the gap between the understanding of TiA and the actual human user interaction behavior which does not appear to have a clear mapping from TiA levels. We argue in this article that, in addition to understanding the influence of changing levels of TiA, understanding the antecedent decision of the human user’s interaction behavior is critical for improving HAI system performance. Decisions have not been explicitly studied in the context of HAI and TiA specifically, but due to the importance of these interaction decisions we reviewed decision making literature and summarized findings that provide a basic understanding of the psychophysiological processes involved in decision making. We are particularly interested in value based decision making because, just as in the case of TiA, if there is no risk, the behavior ceases to be important. While the value based decision process is not yet fully understood as it relates to interaction behaviors, there is a significant understanding of the underlying psychophysiological processes and correlates. This knowledge can be used to advance hypotheses that define a research path aimed at achieving mitigation of human user interaction behaviors.

## Author Contributions

KD: completed literature review and steered review towards current domain; main contributing author. JSM: final approval authority, guided literature review, advanced concepts germane to content, significant contribution to authorship. ARM: advised in fundamental concepts regarding BCI, HCI, and HAI; also reviewed, edited, and approved successive steps in the process. JRL: provided expertise in the cognitive neuroscience of trust in automation; also reviewed, edited and approved successive review and writing processes.

## Conflict of Interest Statement

The authors declare that the research was conducted in the absence of any commercial or financial relationships that could be construed as a potential conflict of interest. The reviewer DM and handling Editor declared their shared affiliation, and the handling Editor states that the process nevertheless met the standards of a fair and objective review.
